# Effect of hydroxylpropyl-β-cyclodextrin on Solubility of Carvedilol

**DOI:** 10.4103/0250-474X.41470

**Published:** 2008

**Authors:** B. D. Shewale, N. P. Sapkal, N. A. Raut, N. J. Gaikwad, R. A. Fursule

**Affiliations:** H. R. Patel Women’s Collage of Pharmacy, Karwand Naka (Shirpur)- 425 405, India

**Keywords:** Carvedilol, hydroxypropyl-β-cyclodextrin, solubility, bioavailability, pH

## Abstract

The present study was undertaken to examine the effect of pH and concentration of hydroxypropyl-β-cyclodextrin on the solubility of carvedilol as it shows pH-dependent solubility. The equilibrium solubility of carvedilol in a series of solutions of varying pH (from 1.2 to 11) was determined and compared with the equilibrium solubility of carvedilol in the presence of 20% hydroxypropyl-β-cyclodextrin at same pH values. It was observed that solubility of protonated form is more than neutral molecule. Hydroxypropyl-β-cyclodextrin resulted in increased solubility at all the pH. But inclusion in the cavity of hydroxypropyl-β-cyclodextrin might depend upon charge state of the molecule. So it can be concluded that solubility of carvedilol, can be increased either by the addition of hydroxypropyl-β-cyclodextrin or by adding pH lowering agents. But both these methods if are to be used together, pH should be selected carefully.

Carvedilol, (2 RS)-1-(9H-Carbazol-4-yloxy)-3-[[2-(2-methoxyphenoxy)ethyl]-amino]-2-propanol, is basic compound with pKa value of 7.8^1^. Carvedilol is used for the treatment of mild to moderate hypertension. Carvedilol is a nonselective beta-adrenoreceptor antagonist and a α_1_-adrenoreceptor antagonist and a vasodilator. The vasodilatory actions of carvedilol result primarily from α_1_-adrenoreceptor blockade, whereas the α/β-adrenoreceptor blocking activity of carvedilol prevents reflex tachycardia when used in the treatment of hypertension^2^. The multiple actions of carvedilol are responsible for the antihypertensive efficacy of the drug. Apart from being an antihypertensive agent it is also used in other cardiovascular disorders such as angina pectoris, cardiac arrhythmias and myocardial infarction^3^. Because of these therapeutic effects it has emerged as one of the important and promising drug substances for cardiovascular diseases, especially due to the noticeable improvement of survival rates in patients with chronic cardiac insufficiency. But the major problem with this useful drug is its limited solubility in water. This limits not only its bioavailability to 25-35% but also formulation into desired dosage forms.

Hydroxypropyl-β-cyclodextrin (HP-β-CD) has been used in improving the aqueous solubility of a variety of compounds^4^. It is a cyclic oligosaccharide containing seven D-(+)-glucopyranose units, with an average of one hydroxypropyl group per unit. The circular arrangement of the glucose units produces a torus-shaped molecule and CH_2_ groups and ether linkages of the molecule face the hollow interior of the configuration results in a nonpolar cavity and a polar exterior. When a compound with appropriate geometry and HP-β-CD are in the same solution, the non polar aromatic portions of the compound tend to enter the nonpolar interior of the HP-β-CD molecule. This complexation isolates the aromatic portion of the molecule from the water, thereby increasing its aqueous solubility.

Special interest in this study is due to the fact that the carvedilol molecule contains two well-separated benzene groups which may individually complex with HP-β-CD. Because carvedilol is a weak base, the charge state of the drug molecule may influence complex formation, and vice versa. Therefore, the effect of pH on complexation was also examined.

HP-β-CD was obtained as a gift sample from Zim laboratories Ltd. (India). Carvedilol was purchased from Ultratech (India). All other chemicals were of analytical grade and used without further purification. All pH measurements were performed using Elico pH meter (model-140) that was calibrated using pH 4, 7, and 10 standard buffers. Carvedilol concentrations were determined using Shimadzu UVPC 2401 spectrophotometer at 285 nm.

Initially the solubility of carvedilol as a function of pH was studied. A series of buffer solution from pH range 1.2 to 11 were prepared. Carvedilol was added in sufficient quantity to saturate each solution. To avoid change in concentration due to evaporation, solution vials were sealed with Teflon lined screw caps and wrapped with paraffin. All solutions were then placed on a test tube rotator and checked daily for the saturation if necessary pH was adjusted. To ensure the attainment of equilibrium all solutions were rotated for one week. After that the solutions were diluted suitably and determined spectrophotometrically at 285 nm. Similar studies were repeated after the addition of 20% HP-β-CD to the series of buffer solutions.

To explore the effect of cyclodextrin concentration on the solubility of carvedilol, phase solubility studies were performed^5^. A series of solutions containing varying concentrations of HP-β-CD (1% to 40%) in pH 7.4 buffer were prepared. Carvedilol was added to each solution in sufficient quantity to ensure saturation, and previously described procedure was used for determining effect of HP-β-CD concentration on the solubility of carvedilol.

Carvedilol contains a α-hydroxyl secondary amine, with a pKa of 7.8. It exhibits pH dependent solubility. The pH dependence of the complexation of carvedilol with HP-β-CD was investigated on the basis of solubility/pH profiles. (fig. 1) shows the solubility profile of carvedilol in the presence and absence of HP-β-CD, as a function of pH. As can be seen, both in the presence and absence of HP-β-CD, carvedilol exhibited pH dependent solubility. Its solubility increases with decreasing pH and then starts decreasing after pH 4. At lower pH values protonated form of carvedilol and its salt generated *in situ* will determine its solubility. The hydrochloride salt generated in-situ in an acidic medium might be less soluble in this medium than the protonated carvedilol itself. At basic pH as the pH increases from 9.2 to 11 its solubility remains more or less constant (2.5±0.5 μg/ml).

**Fig 1:**
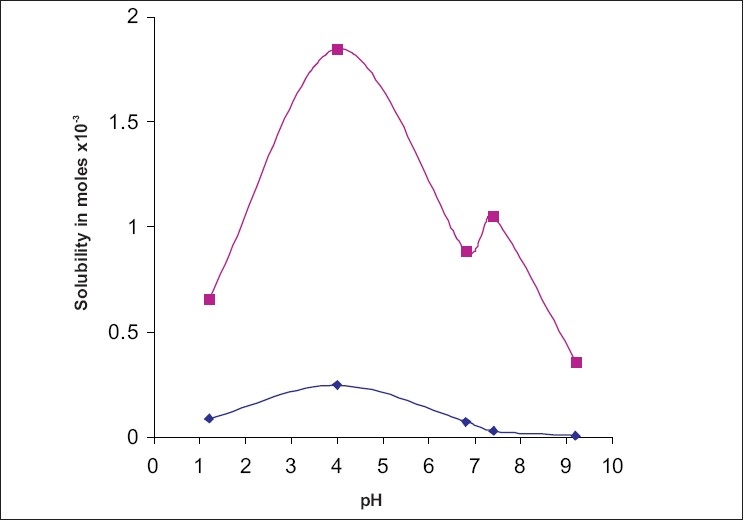
Solubility of carvedilol as a function of pH. Solubility of carvedilol without HP–β–CD (—◆—) and in the presence of 20% HP–β–CD(—■—) at different pH levels.

The addition of HP-β-CD results in a solubility profile as a function of pH similar in shape to that obtained in the absence of complexing agent. However it shows a significant rise in the solubility of carvedilol at all pH values tested. Addition of 20% HP-β-CD increased the solubility to about 8 times at pH 1.2 and 4; 13 times at pH 6.8; 38 times at pH 7.4; 56 times at pH 9.2 and 65 times at pH 11. (fig. 2) shows the percent rise in the solubility of carvedilol by HP-β-CD. This pattern indicates that degree of ionization has a decisive influence on the complexibility, and hence on the solubility of carvedilol at different pH. Both protonated and neutral molecules are not included in the HP-β-CD cavity with same ease. At acidic pH, molecule exists in protonated might be not getting complexed with HP-β-CD. This justifies the limited rise in solubility at acidic pH by HP-β-CD. While at basic pH the major fraction of the molecules exists in unionized form which is hydrophobic. The interior environment of a cyclodextrin cavity is hydrophobic; hence it can entrap unionized form of the molecule which too is hydrophobic^6^. This can be well explained by Henderson Hesselbach equation^7^.

**Fig. 2:**
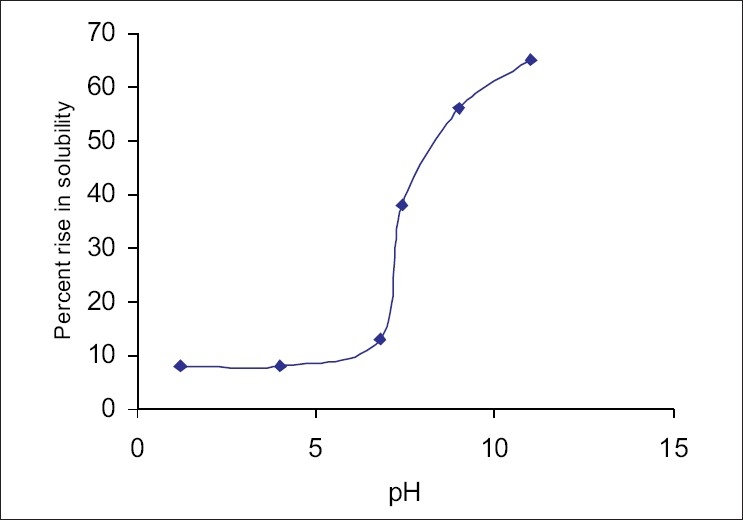
Effect of HP-β-CD on the percent rise solubility of carvedilol

At the acidic pH Carvedilol show appreciable solubility due to its ionization as it is basic in nature. At the acidic pH drug does not form the complex with β-CD due to its ionization, while at the basic pH drug does not get solubilize and remain in its unionized form and get enclosed in the hydrophobic cavity of β-CD, forms the complexation and enhance the solubility at basic pH.

Phase solubility studies of carvedilol were performed at pH 7.4. (fig. 3) shows the changes in the solubility of carvedilol with the increasing concentration of HP-β-CD. It shows a linear rise in solubility. The apparent stability constant was calculated with the assumption of 1:1 stoichiometry and was found to be 4.19×10^4^ at pH 7.4 The value of stability constant indicates that complex is adequately stable and HP-β-CD can be used to improve the aqueous solubility of carvedilol. But since improvement in solubility is not constant at all the pH values, due attention should be paid to this fact, while preparing formulation, and carrying out its bioavailability studies. A suitable acidifying agent must be incorporated to get a steady enhancement in solubility.

**Fig. 3:**
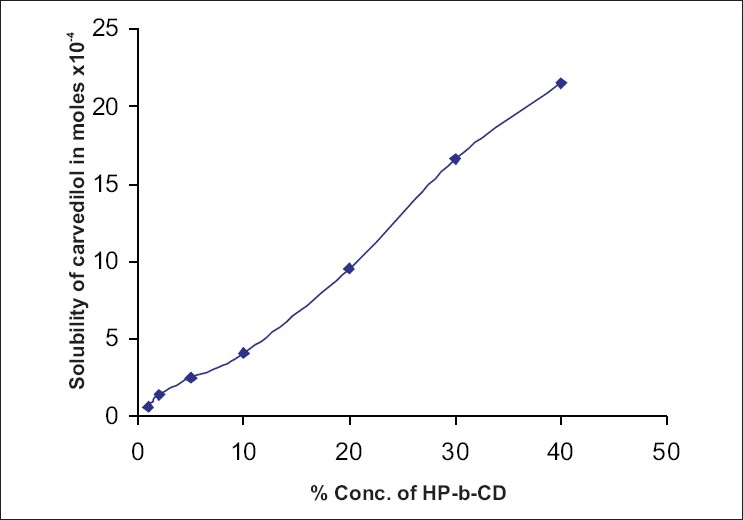
Solubility of carvedilol versus concentration of HP-β-CD at pH 7.4.
